# A child with green urine after a diagnostic enema: Questions

**DOI:** 10.1007/s00467-021-05028-5

**Published:** 2021-03-17

**Authors:** Luisa Cortellazzo Wiel, Giulia Gortani, Davide Zanon, Matteo Bramuzzo, Marco Pennesi, Egidio Barbi

**Affiliations:** 1 University of Trieste, Trieste, Italy; 2 Institute for Maternal and Child Health – IRCCS “Burlo Garofolo”, Trieste, Italy

**Keywords:** Child, Green urine, Recurrent cystitis, Autoimmune gastritis, Cystoscopy, Colonoscopy

## Case report

**Fig. 1:**
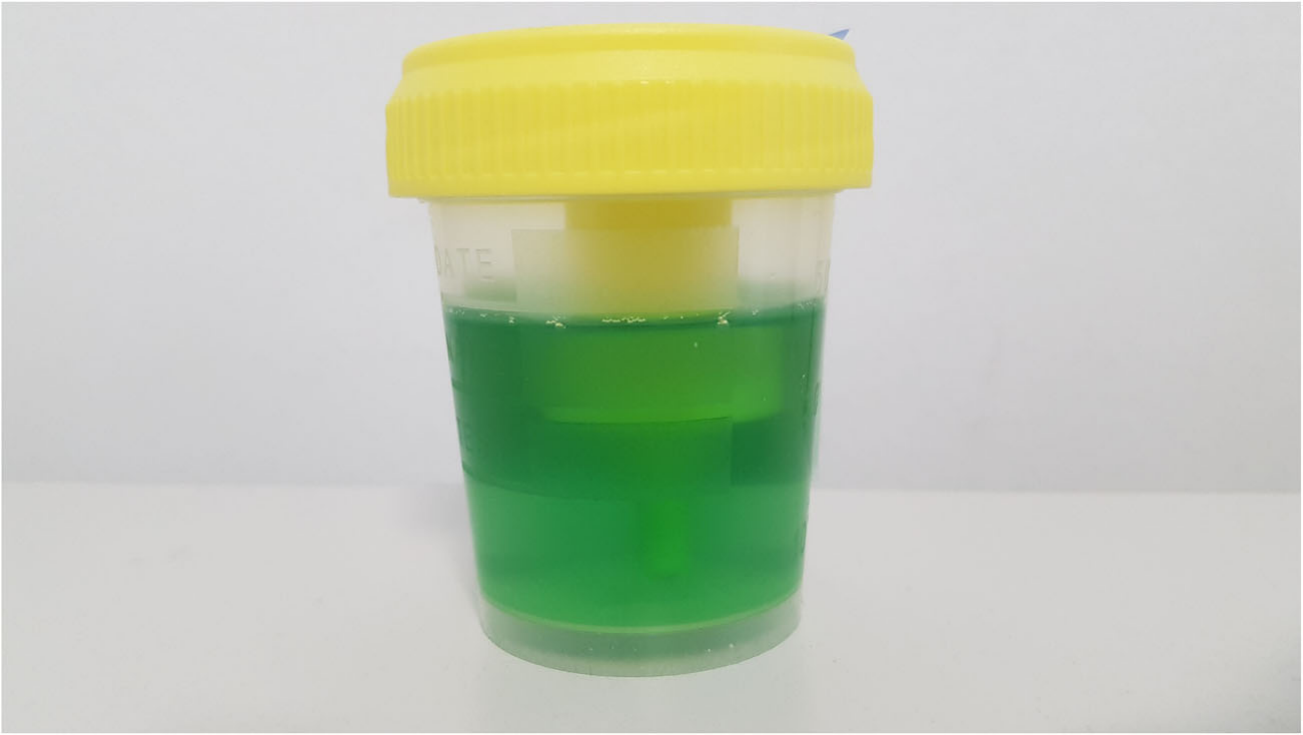
Green urine of the patient

A 12-year-old boy was evaluated for recurrent cystitis due to *Enterococcus faecalis*. The boy had a history of autoimmune gastritis, constipation with encopresis, and primary enuresis. An ultrasound scan ruled out the presence of a urinary tract malformation, showing a mild wall thickening of both the bladder and the rectum. The magnetic resonance imaging study evoked the suspicion of a possible recto-urethral fistula. To confirm this hypothesis, combined cystoscopy and colonoscopy were scheduled, under general anesthesia with sevoflurane, fentanyl, and propofol. Cystoscopy was performed while irrigating the rectum with methylene blue, to assist the detection of any fistulous tract. Despite the instillation of a large amount of dye, the test proved negative. A colonoscopy was eventually performed after washing the rectum, showing no evidence of inflammation. The night following the procedure, the boy referred to voiding bluish urine. During the following day, a greenish hue of the urine was noted (Fig. 1), which gradually faded within a few days. Urinalysis was otherwise unremarkable.

## Questions

1. What is the differential diagnosis for a child with green urine?
2. What diagnostic tests are useful to establish the diagnosis?
3. How would you manage this patient?

## Data Availability

Data sharing is not applicable to this article as no datasets were generated or analyzed during the current study.

